# Dry Eye in Vernal Keratoconjunctivitis: A Cross-Sectional Comparative Study

**DOI:** 10.1097/MD.0000000000001648

**Published:** 2015-10-23

**Authors:** Edoardo Villani, Marika Dello Strologo, Francesco Pichi, Saverio V. Luccarelli, Stefano De Cillà, Massimiliano Serafino, Paolo Nucci

**Affiliations:** From the Department of Clinical Sciences and Community Health, University of Milan, Eye Clinic San Giuseppe Hospital, IRCCS Multimedica, Milan (EV, MDS, FP, SVL, MS, PN); Eye Clinic Azienda Ospedaliero-Universitaria Maggiore della Carità, Novara (SDC); and Eye Clinic San Paolo Hospital, Milan, Italy (SDC).

## Abstract

The purpose of this comparative cross-sectional study was to investigate the use of standardized clinical tests for dry eye in pediatric patients with active and quiet vernal keratoconjunctivitis (VKC) and to compare them with healthy children.

We recruited 35 active VKC, 35 inactive VKC, and 70 age-matched control healthy subjects. Each child underwent a complete eye examination, including visual analog scale symptoms assessment, biomicroscopy, fluorescein break-up time (BUT), corneal fluorescein and conjunctival lissamine green staining, corneal esthesiometry, Schirmer test with anesthetic, and meibomian glands inspection and expression.

Active VKC patients showed significantly increased symptoms and signs of ocular surface disease, compared with the other 2 groups. Inactive VKC patients, compared with control subjects, showed increased photophobia (*P* < 0.05; Mann-Whitney *U* test), conjunctival lissamine green staining and Schirmer test values, and reduced BUT and corneal sensitivity [*P* < 0.05 by analysis of variance (ANOVA) least significant difference posthoc test for BUT and Schirmer; *P* < 0.001 by Mann-Whitney *U* test for lissamine green staining and corneal sensitivity].

Our results confirm the association between VKC and short-BUT dry eye. This syndrome seems to affect the ocular surface in quiescent phases too, determining abnormalities in tear film stability, epithelial cells integrity, and corneal nerves function. The very long-term consequences of this perennial mechanism of ocular surface damage have not been fully understood yet.

## INTRODUCTION

Pediatric dry eye may be associated with several congenital, autoimmune, and inflammatory disorders, but it has not been investigated as well as in adults and its diagnosis is often overlooked.^1^ Twenty years ago, researchers from the Keio University of Tokyo hypothesized an overlap between dry eye and allergic conjunctivitis,^2,3^ and more recently, some reports have shown the synergic effect of these 2 conditions in affecting tear film dynamics and stability and ocular surface homeostasis.^4–7^ This topic is of exceptional clinical relevance in children, who are more susceptible to the epithelial damage related to prolonged ocular surface inflammation.^8^

Vernal keratoconjunctivitis (VKC) is a severe pediatric, sight-threatening allergic eye disease, which impairs the child's quality of life and can lead to severe ocular complications.^9^ Children with VKC present with severe ocular symptoms, frequently with giant papillae on the upper tarsal conjunctiva (cobblestoning appearance), and/or with gelatinous infiltrations around the limbus surrounding the cornea (Horner–Trantas dot). If untreated, ocular surface remodeling leads to corneal ulcers and scars.^9^

Both epidemiological^10^ and experimental^4,5^ studies have reported the association between severe ocular allergy and dry eye, but clinical management of VKC is usually focused just on classical symptoms and signs of active disease and on the risk of severe corneal involvement.

The purpose of this comparative cross-sectional study was to investigate the results of standardized clinical tests for dry eye in pediatric patients with active and quiet VKC and to compare them with healthy children.

## METHODS

### Design and Participants

This comparative cross-sectional study was performed at a tertiary referral center for pediatric ophthalmology (University Eye Clinic of San Giuseppe Hospital, Milan, Italy). The protocol was approved by the local IRB, and the study was conducted according to the tenets of the Declaration of Helsinki. Written informed consent was obtained by the parents of each child.

From December 2014 to February 2015, we consecutively recruited 35 patients with quiet VKC (defined as no symptoms or mild discomfort, and absence of corneal abnormalities at the time of the examination) and 35 age-matched control subjects (winter C). From April 2015 to May 2015, we consecutively recruited 35 patients with active VKC (defined as moderate to severe ocular discomfort including photophobia, papillae on the upper tarsal conjunctiva, or limbal Horner–Trantas dots clearly recognizable at the time of the examination) and 35 age-matched control subjects (spring C).

No patient enrolled as quiet VKC was included in the subsequently recruited active VKC group. Inclusion criteria for each enrolled patient were age less than 16 years; history and previous diagnosis of VKC; and the willingness and capability of the child to be compliant with tests execution and informed consent given by parents. Exclusion criteria were systemic diseases (other than atopy) or therapies with known effect on the ocular surface; history of Stevens–Johnson syndrome; chemical, thermal, or radiation injury to the eye; previous ophthalmic surgery; active VKC with corneal shield ulcer, and use of topical drugs (other than mast-cells stabilizers or dual-acting eyedrops) in the 4 weeks before examination.

Healthy control subjects were recruited among children attending our outpatient clinic for refraction test.

## PROCEDURES

Each recruited child underwent a complete eye examination, including symptoms assessment, biomicroscopy, fluorescein break-up time (BUT), corneal fluorescein staining and conjunctival lissamine green staining, corneal esthesiometry, Schirmer test with anesthetic, meibomian glands (MGs) inspection, and expression.

Quantification of dry eye symptoms was performed using the visual analog scale (VAS). The VAS questionnaire in this study consisted of 5 questions, each of which had an answer scale from 0 (no symptom) to 10 (the worst symptom they could imagine), respectively, for itching, photofobia, dryness, foreign-body sensation, and burning/pain. The first 2 items were grouped in a VKC symptoms score (0–20) and the last 3 items were grouped in a dry eye score (0–30).

Corneal and conjunctival staining were evaluated using the Oxford grading schema^11^ and corneal apex sensitivity was assessed by Cochet–Bonnet nylon thread aesthesiomether.

Meiboscopy of the lower eyelid allowed to assess the degree of MG dropout (score: grade 0, no gland dropout; grade 1, gland dropout in less than half of the inferior tarsus; and grade 2, gland dropout in more than half of the inferior tarsus)^12^ and the presence of MG distortion.^13^ Assessment of obstruction in MG orifices was conducted by applying digital pressure on the upper tarsus, after which the degree of ease in expressing MG secretion (meibum) was evaluated semiquantitatively: grade 0, clear meibum easily expressed; grade 1, cloudy meibum expressed with mild pressure; grade 2, cloudy meibum expressed with more than moderate pressure; and grade 3, meibum not expressed even with firm pressure.^12^

Ocular surface tests were performed in the order suggested by the Diagnostic Methodology Subcommittee of the International Dry Eye WorkShop (2007).^14^

### Statistical Analysis

We determined the sample size in order to assess the average BUT in each VKC group at 5% of type of 1 error and precision of 1 second in either side. On the basis of previously published reports,^8,15^ the standard deviation of BUT in children was hypothesized to be equal to 3. Because of the correlation of measures performed on both eyes and to ensure statistical independence between observations, only 1 randomly chosen eye for each subject was included in this analysis.

After verifying the absence of demographical and clinical differences between winter C and spring C, we considered them as a single control group of healthy 70 eyes.

Quantitative data are expressed as mean ± standard deviation. Differences among groups were assessed by analysis of variance (ANOVA) with least significant difference (LSD) posthoc test for parametric variables and Kruskal–Wallis one-way analysis of variance for non-parametric variables. Mann–Whitney *U* test was used to compare single pairs of groups for nonparametric variables. Percentages were compared by Chi-square test. Correlations were explored by Spearman correlation index.

The minimum criterion for tests of significance was *P* value less than 0.05. Missing data were excluded pairwise. The statistical analysis was conducted with commercial software (SPSS for Windows, ver. 12.0; SPSS Sciences, Chicago, IL).

## RESULTS

Quiet VKC, active VKC, and control groups showed no significant differences in age (9.89 ± 3.12, 8.68 ± 3.67, 9.12 ± 5.21, respectively; *P* = n.s., ANOVA), while the percentage of males was higher in the control group (29%, 31%, 47%, respectively; *P* < 0.001, Chi-square test).

All symptoms assessed by VAS, except dryness, showed significant differences between active VKC and the other 2 groups (*P* < 0.001; Kruskal–Wallis one-way analysis of variance). Photophobia score was significantly higher in quiet VKC than in controls (*P* < 0.05; Mann–Whitney *U* test) (Table 1). All clinical tests, except MG assessment, showed significant differences between active VKC and the 2 other groups (*P* < 0.001; ANOVA for BUT and Schirmer, Kruskal–Wallis for staining and sensitivity). The same clinical tests, except corneal fluorescein staining, showed significant differences between quiet VKC patients and controls too (*P* < 0.05 by LSD posthoc test for BUT and Schirmer; *P* < 0.001 by Mann–Whitney *U* test for lissamine green staining and corneal sensitivity). The examination of MG dropout and expressibility revealed no differences among the groups, while MG distortion was more frequent in both VKC groups than in controls (*P* < 0.001; Chi-square test) (Table 2).

**TABLE 1:**
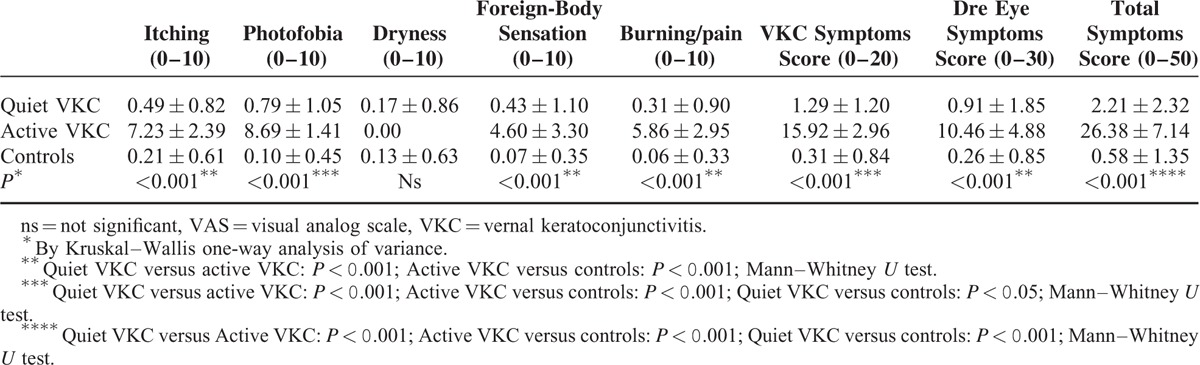
Symptoms Assessed by VAS

**TABLE 2:**
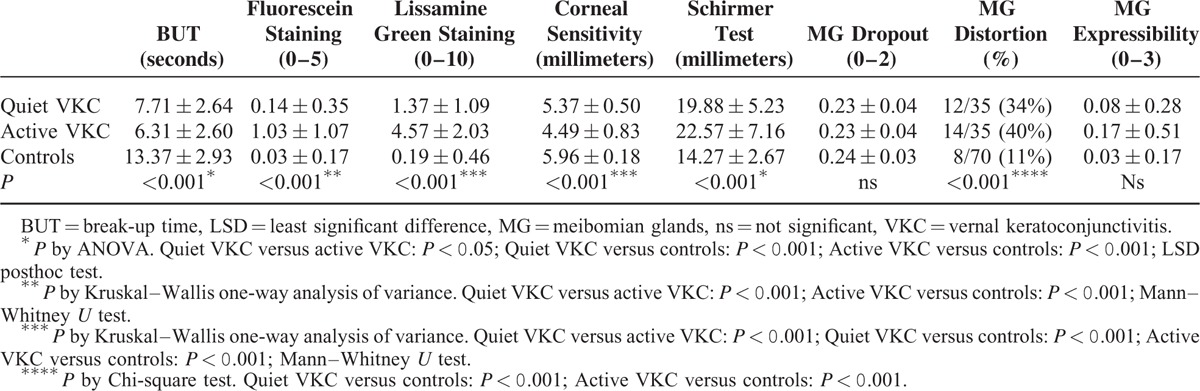
Signs of Dry Eye

No significant correlations were found among the different symptoms. Exploring correlations between symptoms and signs, a positive correlation between photophobia and Schirmer test value emerged (*r* = 0.46, *P* < 0.001; Spearman). Among the clinical signs, significant positive correlations were found between fluorescein and lissamine staining (*r* = 0.54, *P* < 0.001) and between BUT and corneal sensitivity (*r* = 0.56, *P* < 0.001), while a negative correlations were detected between BUT and lissamine green staining (*r* = -0.65, *P* < 0.001).

In VKC patients, comparing symptoms and signs in subjects with and without MG distortion, no significant differences were found.

## DISCUSSION

Previous reports showed tear film disfunction and cytological signs of dry eye in patients with severe ocular allergy.^5,15,16^ In this study, on the basis of the Clinical Grading of Vernal Keratoconjunctivitis proposed by Sacchetti et al,^17^ we defined and compared quiet VKC (grade 0–1) and active VKC (grade 2–3). We think that taking ocular surface alterations into consideration when dealing with VKC may provide new and important information on this disease, which shows a great variability of clinical manifestations with several alternating episodes of improvement or relapse per year.^17,18^

The lack of associations between dry eye signs and symptoms is well known^19^ and recent evidences suggest that children may report less severe symptoms than adult patients.^20^ In our study, active VKC patients reported a wide range of symptoms, characteristic of both severe allergy (itch and photophobia) and dry eye (foreign body sensation and burning/pain). No subjects complained about dryness, probably because of tearing and high tear volume and secretion, as suggested by Schirmer test results. Despite several objective clinical differences between quiet VKC and controls, photophobia was the unique symptom with different values between these 2 groups. According to previous reports,^4,5,15,16^ our active VKC patients had reduced BUT and corneal sensitivity and increased corneal and conjunctival staining and Schirmer values. These findings, together with normal results of MG dropout and expressibility assessment, seem to suggest the presence of a short-BUT dry eye, mainly due to inflammation and mucin changes. As previously reported,^4,5^ inflammation, even in absence of evident aqueous or lipid deficiency, may affect goblet cells, MUC5AC mRNA expression, and corneal nerves, leading to tear function alterations. MGs distortion, significantly more frequent in VKC than in controls, has been previously reported in association with other types of ocular allergy,^13,21^ but its clinical role still needs to be clarified. Our quiet VKC patients, compared with controls, showed reduced BUT and corneal sensitivity and increased conjunctival lissamine green staining. To the best of our knowledge, this is the first study reporting standardized assessment of subclinical tear film dysfunction in quiet VKC. Similar information have been indirectly reported in an in vivo confocal study by Leonardi et al,^22^ who described nerves and epithelial changes in patients with different grades of VKC severity, including mild and paucisymptomatic cases. Our findings of ocular surface alterations in quiet VKC need to be considered in the daily management of these pediatric patients and imply that treating VKC patients to reach a reduction of symptoms and to avoid severe corneal complications may not be enough. Further investigations will be important to understand the very long-term effects of this chronic ocular surface disease.

Although trying to assess ocular surface symptoms in a research on pediatric patients, the absence of standardized and dedicated methods may represent a limitation. Furthermore, children may experience difficulties of comprehension of standardized questionnaires; in order to try to minimize this incomprehension, we decided to use VAS.^20^ Another limitation of this study may be the nonuse of high-tech biomarkes, in addition to standardized clinical tests. In quiet VKC patients, tear and conjunctival cytology,^9^ in vivo confocal microscopy,^23,24^ and tear film proteins quantification^25^ could provide important information on subclinical abnormalities of the ocular surface.

In summary, VKC is a severe form of ocular allergy, and in addition to typical inflammatory tarsal and/or limbal manifestations, it is associated with tear film dysfunction, affecting tear film stability, corneal nerves function, and epithelial cells integrity. These changes seem to persist even in the quiescent phases of the disease, determining a perennial, not yet fully understood, potential mechanism of damage of the ocular surface. A deeper understanding of these mechanisms might lead to hypothesize the need to add to current VKC management, mainly aimed to avoid corneal complications and to control symptoms, measures to prevent the very long-term consequences of protracted, nearly subclinical at alternate stages, ocular surface disease.

## References

[1] Alves M Dias AC Rocha EM Dry eye in childhood: epidemiological and clinical aspects. *Ocul Surf* 2008; 6:44–51. 18264654 10.1016/s1542-0124(12)70104-0

[2] Fujishima H Toda I Shimazaki J Allergic conjunctivitis and dry eye. *Br J Ophthalmol* 1996; 80:994–997. 8976728 10.1136/bjo.80.11.994 PMC505678

[3] Toda I Shimazaki J Tsubota K Dry eye with only decreased tear break-up time is sometimes associated with allergic conjunctivitis. *Ophthalmology* 1995; 102:302–309. 7862418 10.1016/s0161-6420(95)31024-x

[4] Dogru M Okada N Asano-Kato N Atopic ocular surface disease: implications on tear function and ocular surface mucins. *Cornea* 2005; 24 (8 Suppl):S18–S23. 16227818 10.1097/01.ico.0000178741.14212.53

[5] Hu Y Matsumoto Y Dogru M The differences of tear function and ocular surface findings in patients with atopic keratoconjunctivitis and vernal keratoconjunctivitis. *Allergy* 2007; 62:917–925. 17620070 10.1111/j.1398-9995.2007.01414.x

[6] Hom MM Nguyen AL Bielory L Allergic conjunctivitis and dry eye syndrome. *Ann Allergy Asthma Immunol* 2012; 108:163–166. 22374198 10.1016/j.anai.2012.01.006

[7] Bielory L Ocular allergy and dry eye syndrome. *Curr Opin Allergy Clin Immunol* 2004; 4:421–424. 15349042 10.1097/00130832-200410000-00014

[8] Onguchi T Dogru M Okada N The impact of the onset time of atopic keratoconjunctivitis on the tear function and ocular surface findings. *Am J Ophthalmol* 2006; 141:569–571. 16490512 10.1016/j.ajo.2005.09.032

[9] Vichyanond P Pacharn P Pleyer U Vernal keratoconjunctivitis: a severe allergic eye disease with remodeling changes. *Pediatr Allergy Immunol* 2014; 25:314–322. 24438133 10.1111/pai.12197

[10] Bonini S Lambiase A Marchi S Vernal keratoconjunctivitis revisited: a case series of 195 patients with long-term followup. *Ophthalmology* 2000; 107:1157–1163. 10857837 10.1016/s0161-6420(00)00092-0

[11] Bron AJ Evans VE Smith JA Grading of corneal and conjunctival staining in the context of other dry eye tests. *Cornea* 2003; 22:640–650. 14508260 10.1097/00003226-200310000-00008

[12] Shimazaki J Sakata M Tsubota K Ocular surface changes and discomfort in patients with meibomian gland dysfunction. *Arch Ophthalmol* 1995; 113:1266–1270. 7575257 10.1001/archopht.1995.01100100054027

[13] Arita R Itoh K Maeda S Meibomian gland duct distortion in patients with perennial allergic conjunctivitis. *Cornea* 2010; 29:858–860. 20508507 10.1097/ICO.0b013e3181ca3668

[14] Methodologies to diagnose and monitor dry eye disease: report of the Diagnostic Methodology Subcommittee of the International Dry Eye WorkShop (2007). *Ocul Surf* 2007; 5:108–152. 17508118 10.1016/s1542-0124(12)70083-6

[15] Dogru M Gunay M Celik G Aktas A Evaluation of the tear film instability in children with allergic diseases. Cutan Ocul Toxicol. 2015 Feb 19:1–4. [Epub ahead of print]. 10.3109/15569527.2015.1010727 25694171

[16] Dogru M Matsumoto Y Okada N Alterations of the ocular surface epithelial MUC16 and goblet cell MUC5AC in patients with atopic keratoconjunctivitis. *Allergy* 2008; 63:1324–1334. 18782111 10.1111/j.1398-9995.2008.01781.x

[17] Sacchetti M Lambiase A Mantelli F Tailored approach to the treatment of vernal keratoconjunctivitis. *Ophthalmology* 2010; 117:1294–1299. 20382430 10.1016/j.ophtha.2009.11.043

[18] Lambiase A Minchiotti S Leonardi A Prospective, multicenter demographic and epidemiological study on vernal keratoconjunctivitis: a glimpse of ocular surface in Italian population. *Ophthalmic Epidemiol* 2009; 16:38–41. 19191180 10.1080/09286580802573177

[19] Nichols KK Nichols JJ Mitchell GL The lack of association between signs and symptoms in patients with dry eye disease. *Cornea* 2004; 23:762–770. 15502475 10.1097/01.ico.0000133997.07144.9e

[20] Han SB Yang HK Hyon JY Children with dry eye type conditions may report less severe symptoms than adult patients. *Graefes Arch Clin Exp Ophthalmol* 2013; 251:791–796. 22790310 10.1007/s00417-012-2097-2

[21] Arita R Itoh K Maeda S Association of contact lens-related allergic conjunctivitis with changes in the morphology of meibomian glands. *Jpn J Ophthalmol* 2012; 56:14–19. 22109632 10.1007/s10384-011-0103-6

[22] Leonardi A Lazzarini D Bortolotti M Corneal confocal microscopy in patients with vernal keratoconjunctivitis. *Ophthalmology* 2012; 119:509–515. 22176802 10.1016/j.ophtha.2011.09.018

[23] Villani E Mantelli F Nucci P In-vivo confocal microscopy of the ocular surface: ocular allergy and dry eye. *Curr Opin Allergy Clin Immunol* 2013; 13:569–576. 23974688 10.1097/ACI.0b013e328364ec92

[24] Nebbioso M Zicari AM Lollobrigida V Assessment of corneal alterations by confocal microscopy in vernal keratoconjunctivitis. *Semin Ophthalmol* 2015; 30:40–43. 24074451 10.3109/08820538.2013.821508

[25] You J Willcox MD Madigan MC Tear fluid protein biomarkers. *Adv Clin Chem* 2013; 62:151–196. 24772667 10.1016/b978-0-12-800096-0.00004-4

